# Frequencies of Circulating MAIT Cells Are Diminished in Chronic HCV, HIV and HCV/HIV Co-Infection and Do Not Recover during Therapy

**DOI:** 10.1371/journal.pone.0159243

**Published:** 2016-07-14

**Authors:** Michelle Spaan, Sebastiaan J. Hullegie, Boris J. B. Beudeker, Kim Kreefft, Gertine W. van Oord, Zwier M. A. Groothuismink, Marjolein van Tilborg, Bart Rijnders, Robert J. de Knegt, Mark A. A. Claassen, Andre Boonstra

**Affiliations:** 1 Department of Gastroenterology and Hepatology, Erasmus MC, University Medical Center, Rotterdam, the Netherlands; 2 Department of Internal Medicine, Infectious Diseases section Erasmus MC, University Medical Center, Rotterdam, the Netherlands; Karolinska Institutet, SWEDEN

## Abstract

**Objective:**

Mucosal-associated invariant T (MAIT) cells comprise a subpopulation of T cells that can be activated by bacterial products and cytokines to produce IFN-γ. Since little is known on MAIT cells during HCV infection, we compared their phenotype and function in comparison to HIV and HCV/HIV co-infected patients, and determined the effect of IFN-α-based and direct-acting antiviral therapy on MAIT cells of HCV patients.

**Methods:**

Blood samples from patients with chronic HCV (CHCV), virologically suppressed HIV, acute HCV/HIV co-infection (AHCV/HIV) and healthy individuals were examined by flowcytometry for phenotype and function of MAIT and NK cells.

**Results and Conclusions:**

Compared to healthy individuals, the frequency of CD161^+^Vα7.2^+^ MAIT cells was significantly decreased in patients with CHCV, HIV and AHCV/HIV co-infection. CD38 expression on MAIT cells was increased in AHCV/HIV patients. MAIT cells were responsive to IFN-α *in vitro* as evidenced by enhanced frequencies of IFN-γ producing cells. IFN-α-based therapy for CHCV decreased the frequency of IFN-γ^+^ MAIT cells, which was still observed 24 weeks after successful therapy. Importantly, even after successful IFN-α-based as well as IFN-α-free therapy for CHCV, decreased frequencies of MAIT cells persisted. We show that the frequencies of MAIT cells are reduced in blood of patients with CHCV, HIV and in AHCV/HIV co-infection compared to healthy individuals. Successful therapy for CHCV did not normalize MAIT cell frequencies at 24 weeks follow up. The impact of HIV and HCV infection on the numbers and function of MAIT cells warrant further studies on the impact of viral infections and the antimicrobial function of MAIT cells.

## Introduction

Following infection with hepatitis C virus (HCV), hepatocytes are triggered to produce type I and III interferons (IFN), which induce the expression of hundreds of IFN stimulating genes (ISG) with anti-viral activity [1–3]. However, despite the induction of ISG, viral titers increase during acute HCV infection, and in the majority of infected individuals the virus is able to establish a chronic infection of the liver, which indicates that the immune response is ineffective [4, 5]. Besides the induction of ISG, IFN also activates natural killer (NK) cells, T cells and dendritic cells (DCs), and are therefore important immunomodulators [2, 6–9]. Similar as in HCV, type I IFN are produced in large amounts after infection with human immunodeficiency virus (HIV), causing induction of antiviral responses that target every step of the HIV life cycle [9].

In recent years, our understanding of Mucosal-Associated Invariant T (MAIT) cells in chronic HIV infection has increased substantially. Most MAIT cells are CD8^+^ or double negative for CD4 and CD8, and characterized by the expression of CD161 and the invariant T cell receptor (TCR) Vα7.2 that recognizes vitamin metabolites presented by MR1, a MHC class I related protein, on the surface of antigen-presenting cells [10, 11]. MAIT cells are also activated by IL-12 and IL-18 in an MR1-independent manner [12]. MAIT cells are abundant in human blood (1–10% of CD8^+^ T cells) and are known for their antimicrobial activity to bacteria and yeast in the gut and lungs [13, 14] via release of cytokines and cytotoxic enzymes [10]. Interestingly, MAIT cells are reduced in peripheral blood and lymph nodes of patients with chronic HIV infection, and their cytokine production and cytolytic functions are severely affected which has been suggested to be the result of exhaustion. Importantly, the loss and dysfunction of MAIT cells are not recovered after successful combination antiretroviral therapy (cART) therapy [15–22]. It has been suggested that the functional impairment and numerical decline of MAIT cells contributes to the high incidence of bacterial infections observed in HIV patients [18]. At the moment it is unclear what causes the depletion of MAIT cells in HIV infection. Similar findings were reported recently in patients chronically infected with HCV where the MAIT cell numbers in blood were severely reduced during persistent infection [23]. Also in chronic HCV, successful HCV clearance by IFN-free therapy does not result in normalization of MAIT cell numbers.

Because little information is available on the role of MAIT cells in HCV infection, we examine in this study the impact of HCV infection on MAIT cells. In addition, we investigate the consequence of IFN-α exposure on NK cells and MAIT cells during IFN-α based therapy for CHCV and acute-HCV/HIV co-infection.

## Materials and Methods

### Patients and healthy subjects

Heparinized blood was collected from 33 patients with chronic HCV (CHCV) infection, 9 acute HCV patients with cART-suppressed HIV (AHCV/HIV), 10 patients with cART-suppressed HIV mono-infection and 12 healthy subjects. The patient characteristics are listed in table 1. 33 CHCV patients were treated in 4 different historical treatment regimens, and blood was collected at multiple time-points. In cohort 1, 11 patients were treated with pegylated-IFN-alpha-2a (PegIFN-α) and ribavirin for 24 or 48 weeks, according to HCV genotype (NCT00422838, [24]). Patients in cohort 2 (n = 11) were treated with telaprevir, PegIFN-α and ribavirin for 24 or 48 weeks, according to their fibrosis level and previous treatment response to PegIFN-α and ribavirin. Consistent with international guidelines [25], patients were treated with telaprevir, PegIFN-α and ribavirin for the first 12 weeks, and continued treatment consisting of PegIFN-α and ribavirin only (NCT01641094). In this treatment cohort, naïve patients, patients with a partial response (>2log drop in viral load) to previous IFN-based therapy, and patients without cirrhosis were treated for 24 weeks when HCV RNA was undetectable (<15 IU/l) at week 4 and 12 during therapy. In cohort 3, 5 patients were treated with daily daclatasvir and twice daily asunaprevir for 24 weeks (NCT02282709, [26]). In cohort 4, 6 patients were treated with sofosbuvir and daclatasvir with or without ribavirin for 12 or 24 weeks according to the international guidelines (AASLD/IDSA Recommendations for testing, managing, and treating hepatitis C 2015 (hcvguidelines.org) and EASL: recommendations on treatment of hepatitis C 2015). The selection of the treatment regimen was solely made by the treating physician. For patients with CHCV, blood was collected at baseline, week 4, week 12 during therapy and 24 weeks after end of therapy in all 4 cohorts. Patients with acute HCV and cART-suppressed HIV were treated in cohort 5 (n = 9). Patients were treated within 26 weeks after HCV infection with 12-weeks boceprevir, PegIFN-α and ribavirin (NCT01912495). Blood was collected at baseline and at week 4 during therapy. Patients with cART-suppressed HIV mono-infection (n = 10) and 22 healthy subjects (67% male, average age: 54 (range 42–70)) were included as controls. The institutional ethical review board of the Erasmus Medical Center approved the protocols, and written informed consent was obtained from all individuals.

**Table 1:** Patient characteristics.

	All CHCV	CHCV	CHCV	CHCV	CHCV	AHCV/HIV	HIV
		cohort 1	cohort 2	cohort 3	cohort 4	cohort 5	
Treatment strategy		PegIFN/riba	PegIFN/riba telaprevir	Asunaprevir daclatasvir	Sofosbuvir daclatasvir	PegIFN/riba boceprevir cART	cART
Number	33	11	11	5	6	9	10
Gender (% male)		73	82	80	67	100	100
Age (mean, yrs)		45 (27–60)	50 (25–61)	52 (43–66)	59 (36–70)	40 (23–58)	49 (27–65)
HCV RNA(mean, IU/ml)		4.6x10^6^ (3.7x10^2^-2.7x10^7^)	2.90x10^6^ (3.5x10^4^-8.2x10^6^)	1.2 x10^6^ (1.6x10^5^-2.3x10^6^)	2.7 x10^6^ (8.3x10^4^-5.3x10^6^)	0.3 x10^6^ (2.0x10^2^-1.9x10^6^)	
ALT (mean, U/l)		79 (34–164)	70 (29–140)	188 (94–269)	126 (24–196)	324 (33–1070)	
Fibrosis (%) 0-1/2/3/4/n.d.		30/50/20/0/0	27/18/36/18/0	80/0/0/20/0	0/0/33/67		
SVR (%)		82	82	100	100	100	
HCV genotype (%) 1/2/3/4		36/18/46/0	100/0/0/0	100/0/0/0	83/17/0/0	100/0/0/0	
HIV Load <20 geq/ml (%)						100	80
CD4 (mean, x10^9^/ml)						0.87 (0.64–1.37)	0.67 (0.47–0.96)

### Analysis of cell surface molecule expression by flow cytometry

Peripheral blood mononuclear cells (PBMC) were isolated from venous blood by (Ficoll-Paque^™^ plus, Amersham) and frozen at -150°C. PBMC were thawed and washed with RPMI 1640 (Lonza) with 10% FCS (Lonza). For flow cytometry, 500,000 PBMC were used for each staining. Cells were stained with anti-CD38-PerCp-eFluor710 (HB7), anti-CD3-PE-Cy7 (UCHT1), anti-CD161-Pacific Blue (HP-3G10, all eBiosciences), anti-CD4-APC-H7 (SK3, BD Biosciences), anti-TCR Vα7.2-PE (3C10, Biolegend), anti-CD56-APC (N901, Beckman) and live/dead marker (Aqua, Life technologies) for 20 minutes at 4°C in the dark. Positivity for CD38 was determined by setting the gates using internal negative cells. Cells were washed and marker expression was detected by flow cytometry (Canto-II, BD). MAIT cells were defined as CD3^+^CD161^+^Vα7.2^+^ cells within the lymphocyte gate and NK cells were defined as CD3^-^CD56^+^ cells within the lymphocyte gate. Data was analyzed using FlowJo version 10.1 (Tree Star Inc).

### Analysis of intracellular cytokines by flow cytometry

The percentage of cells producing IFN-γ was measured by flowcytometry using various stimuli. For each condition, 500,000 cells were stimulated in a 24 wells plate with IL-12 (0.25 ng/ml, Miltenyi), IL-18 (50 ng/ml, MBL) and IFN-α (2500 IU/ml, Intron-A, Merck). For all conditions, cells were stimulated for 16 hours at 37°C at 5% CO_2_. Brefeldin A (10 μg/ml, Sigma) was added and the cells were incubated for another 3 hours. Cells were stained with anti-CD3-PerCp-Cy5.5 (UCHT1), anti-CD4-APC-H7 (SK3, both BD biosciences), anti-CD69-PE-Cy7 (TPI.55.3), anti-CD161-PB (HP-3G10, both eBiosciences), anti-TCR Vα7.2-PE (3C10, Biolegend), anti-CD56-APC (N901, Beckman) and Live/ dead marker (Aqua, Life technologies). Cells were fixed, permeabilized and stained with anti-IFN-γ-FITC (25723.11, BD Biosciences). Cytokine-producing cells were detected by flow cytometry (Canto-II, BD). Quadrants were set on low or absent expression on lineage negative cells. Data was analyzed using FlowJo version 10.1 (Tree Star Inc).

### Statistics

Statistical comparison was tested using the Kruskal-Wallis and Mann-Whitney test for unpaired non-parametric analyses and the Paired student t-test for paired observations. A *p* value ≤ 0.05 was considered significant. All data was analysed using GraphPad Prism (GraphPad Software).

## Results

### MAIT cells are decreased in patients with chronic HCV, HIV and AHCV/HIV co-infection compared to healthy controls

**Fig 1:**
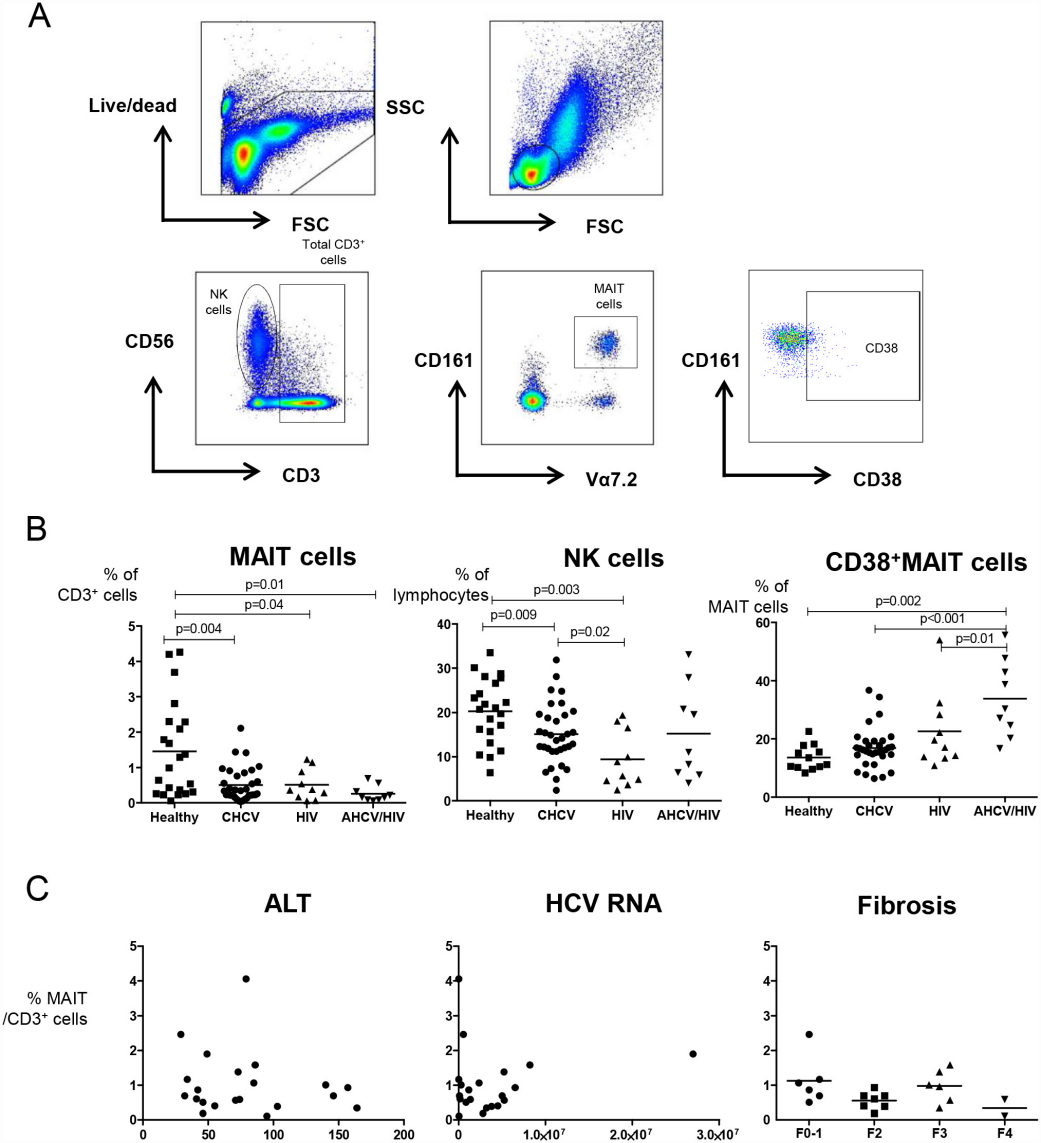
Frequencies of MAIT cells are decreased in patients with CHCV, HIV and AHCV/HIV co-infections compared to healthy individuals. (A) Representative dot plots and gating strategy of CD3^+^CD161^+^ Vα7.2^+^ MAIT cells and CD3^-^CD56^+^ NK cells. (B) The percentage of MAIT cells within the CD3^+^ T cell population, NK cells within the lymphocyte population, and CD38^+^MAIT cells within the total MAIT cell population of healthy individuals (n = 22 for frequencies of MAIT and NK cells; n = 12 for frequencies of CD38^+^ MAIT cells), chronic HCV (CHCV) patients (n = 33), cART-suppressed HIV (HIV) patients (n = 10) and patients with acute HCV/HIV co-infection (AHCV/HIV) (n = 9). (C) Frequency of MAIT cells within the CD3^+^ T cell population in CHCV patients do not correlate with ALT, HCV RNA or fibrosis score levels. No association between the frequency of MAIT cells and ALT levels (r-value: -0.185, p = 0.42) or viral load (r-value: 0.019, p = 0.93) was observed. Statistical comparison was tested using Mann-Whitney test for unpaired analyses. Spearman’s correlation test was used for the first two panels of Fig 1C.

We investigated whether MAIT cells are affected during chronic viral infections and compared them to NK cells. For this purpose, the frequencies of CD3^+^CD161^+^Vα7.2^+^ MAIT cells and CD3^-^CD56^+^ NK cells were determined in peripheral blood of patients with CHCV, HIV, AHCV/HIV and healthy individuals by flow cytometry (Fig 1A and 1B). A significant decrease was observed in the frequencies of MAIT cells in CHCV, HIV and AHCV/HIV patients as compared to healthy controls (p = 0.004, p = 0.04 and p = 0.01, respectively, Fig 1B). No differences were observed in frequencies of NK cells in patients with AHCV/HIV co-infection compared to healthy controls [27–30], whereas NK cells in CHCV and HIV mono-infected patients were decreased compared to healthy controls (p = 0.009 and p = 0.003, respectively) (Fig 1B). Next, we determined the expression of the activation marker CD38 on MAIT cells and observed that patients with AHCV/HIV co-infection had increased frequencies of activated MAIT cells in peripheral blood compared to HIV, CHCV and healthy individuals (p = 0.01, p<0.001, p = 0.002 respectively, Fig 1B). No association between the frequency of MAIT cells and ALT levels (r-value: -0.185, p = 0.42) or viral load (r-value: 0.019, p = 0.93) was observed (Fig 1C). Also, stratification of patients with CHCV of their fibrosis stage showed similar frequencies of MAIT cells (Fig 1C).

### Frequency of IL12/IL-18 induced IFN-y producing MAIT cells does not differ between patients with CHCV, HIV, AHCV/HIV co-infection or healthy individuals

**Fig 2:**
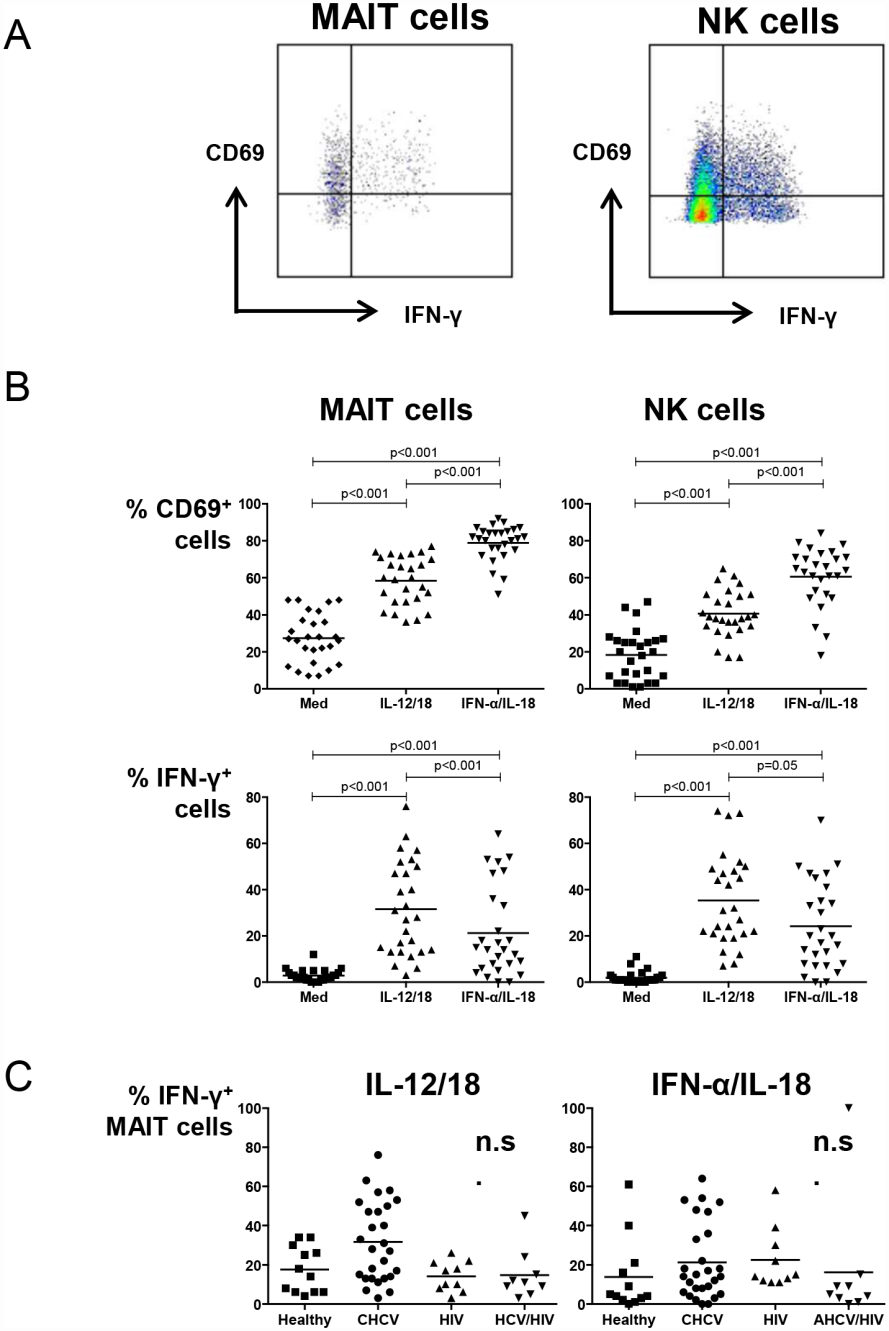
MAIT cells function does not differ between patients with chronic HCV, HIV and AHCV/HIV co-infection. (A) Representative dot plots of CD69 expression and IFN-γ production by MAIT and NK cells in CHCV after stimulation with IL-12/IL-18. (B) Frequency of CD69 expression and IFN-γ producing MAIT cells and NK cells in CHCV patients after 19 hours stimulation with medium, IL-12/IL-18 and IFN-α/IL-18. (C) Frequency of IFN-γ producing MAIT cells after various stimuli between healthy individuals, patients with CHCV, HIV and AHCV/HIV co-infection. Statistical comparison was tested using paired T test (2B) and Mann-Whitney test (2C).

Next, we determined the ability of MAIT cells from the various patient groups to respond to IL-12/18 as well as to IFN-α/IL-18 and evaluated CD69 surface expression and production of IFN-γ. A representative dot plot and gating strategy after stimulation of CHCV peripheral blood with IL-12/18 is shown in Fig 2A. As shown in Fig 2B, CD69 expression on both MAIT cells and NK cells was upregulated upon stimulation with IL-12/18 and IFN-α/IL-18, as compared to medium control conditions. The frequency of IFN-γ producing MAIT cells was significantly increased upon stimulation with IL-12/18. Importantly, IFN-α was also found to be a potent activator of MAIT cells (Fig 2B). Next, we determined the function of MAIT cells in different viral infections and observed that MAIT cells are equally capable to become activated and produce IFN-γ upon stimulation with IL-12/18 and IFN-α/IL-18 in CHCV, HIV, AHCV/HIV co-infection and healthy individuals (Fig 2C).

### IFN-based therapy for chronic HCV reduces the frequency of IFN-γ producing MAIT cells upon IL-12/IL-18 stimulation

**Fig 3:**
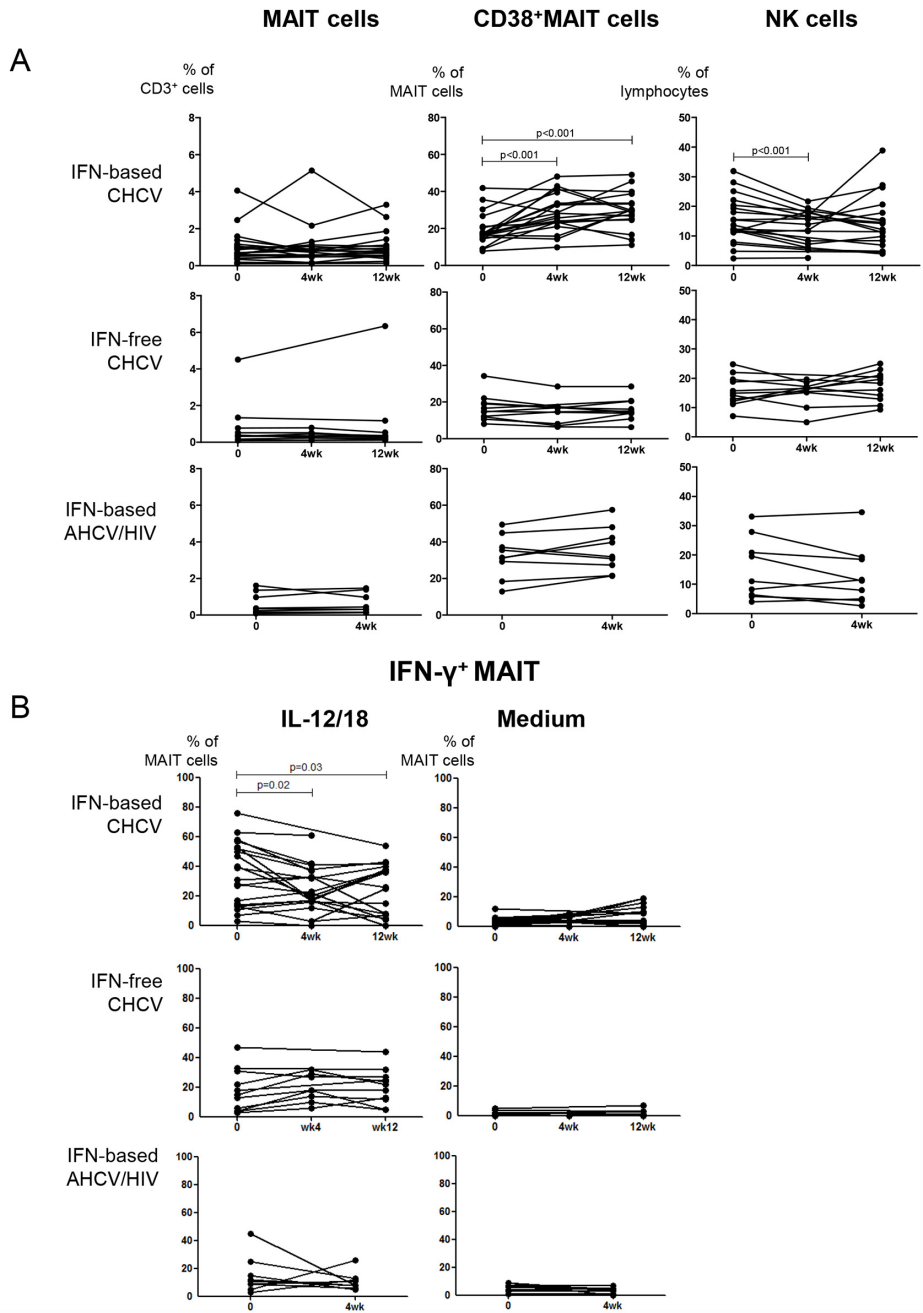
IFN-based therapy for chronic HCV increases expression of CD38 on MAIT cell but reduces MAIT cell function. (A) Frequency of MAIT, CD38^+^MAIT cells and NK cells during IFN-based therapy for CHCV (cohorts 1 and 2, n = 22), IFN-free therapy for CHCV (cohort 3 and 4, n = 11) and IFN-based therapy for AHCV/HIV co-infection (cohort 5, n = 9). At week 12 during therapy for CHCV and at week 4 during therapy for AHCV/HIV, all patients were HCV RNA negative. (B) Frequency of IFN-γ producing MAIT cells after stimulation with IL-12/18, and medium during IFN-based and IFN-free therapy for CHCV infection (baseline, 4wk, 12wk), and during IFN-based therapy for AHCV/HIV (baseline, 4wk). The baseline frequencies of CD38^+^ MAIT cells and IFN-γ^+^ MAIT cells of the patient groups receiving IFN-based and IFN-free therapy were similar (Mann Whitney test). All other statistical comparisons were tested using Wilcoxon matched pairs T test.

Fig 2B shows that IFN-α is a potent stimulator of MAIT cells *in vitro*. Since IFN-α has potent antiviral activity against HCV, we determined whether IFN-α-based therapy for CHCV activates MAIT cells and affects MAIT cell frequencies in CHCV and AHCV/HIV co-infection. Twenty-two CHCV patients were treated with IFN-based therapy with (n = 11) or without (n = 11) telaprevir. All patients were HCV RNA negative at week 12 during therapy. We observed that IFN-α-based therapy did not alter MAIT cell frequencies, but did increase CD38 expression on MAIT cells (p<0.001, Fig 3A, upper panels). No difference was observed between treatment with or without addition of telaprevir (S1 Fig). The NK cell frequency was reduced early during IFN-based therapy (p<0.001), but this was not sustained at week 12 (Fig 3A, upper panels). Recently, IFN-free therapy became available for clinical use and has substituted IFN-α-based therapy because of higher SVR rates and reduced side-effects. Eleven CHCV patients were treated with an IFN-free regimen and we determined if the observed effects on MAIT cells were due to a direct effect of IFN-α or to viral load decline. In all patients, HCV RNA titers were undetectable after 4 weeks of treatment; however, no effect was observed on MAIT cell frequency or activation (Fig 3A, middle row). In addition, no effect was observed on NK cell frequencies (Fig 3A, middle panels). These data suggest that the increased frequencies of CD38-expressing MAIT cells in CHCV patients is the consequence of exposure to IFN-α, rather than of viral load decline.

Patients with AHCV/HIV co-infection were treated with a combination of boceprevir, PegIFN-α and ribavirin; all patients were HCV RNA negative at week 4 during therapy. IFN-α-based therapy did not alter MAIT cell or NK cell frequencies in this patient population (Fig 3A, lower panels). The enhanced expression of CD38 on MAIT cells of patients with AHCV/HIV co-infection (Fig 1) was not further increased during therapy (Fig 3A, lower panels).

To investigate whether MAIT cell function was altered during therapy for HCV, cells were stimulated with IL-12/18. Surprisingly, although IFN-α-based therapy for CHCV caused increased expression of activation marker CD38 on MAIT cells as shown in Fig 3A, a decrease in the frequency of IFN-y producing MAIT cells was observed upon stimulation with IL-12/18 during therapy compared to medium (p = 0.02 at week 4, p = 0.03 at weeks 12; Fig 3B, upper panels). Neither in CHCV patients treated with IFN-free therapy, nor in patients with AHCV/HIV co-infection, therapy altered the frequencies of IFN-γ producing MAIT cells (Fig 3B, middle and lower panels).

### Successful therapy for chronic HCV does not restore MAIT cell frequency 24 weeks after therapy

**Fig 4:**
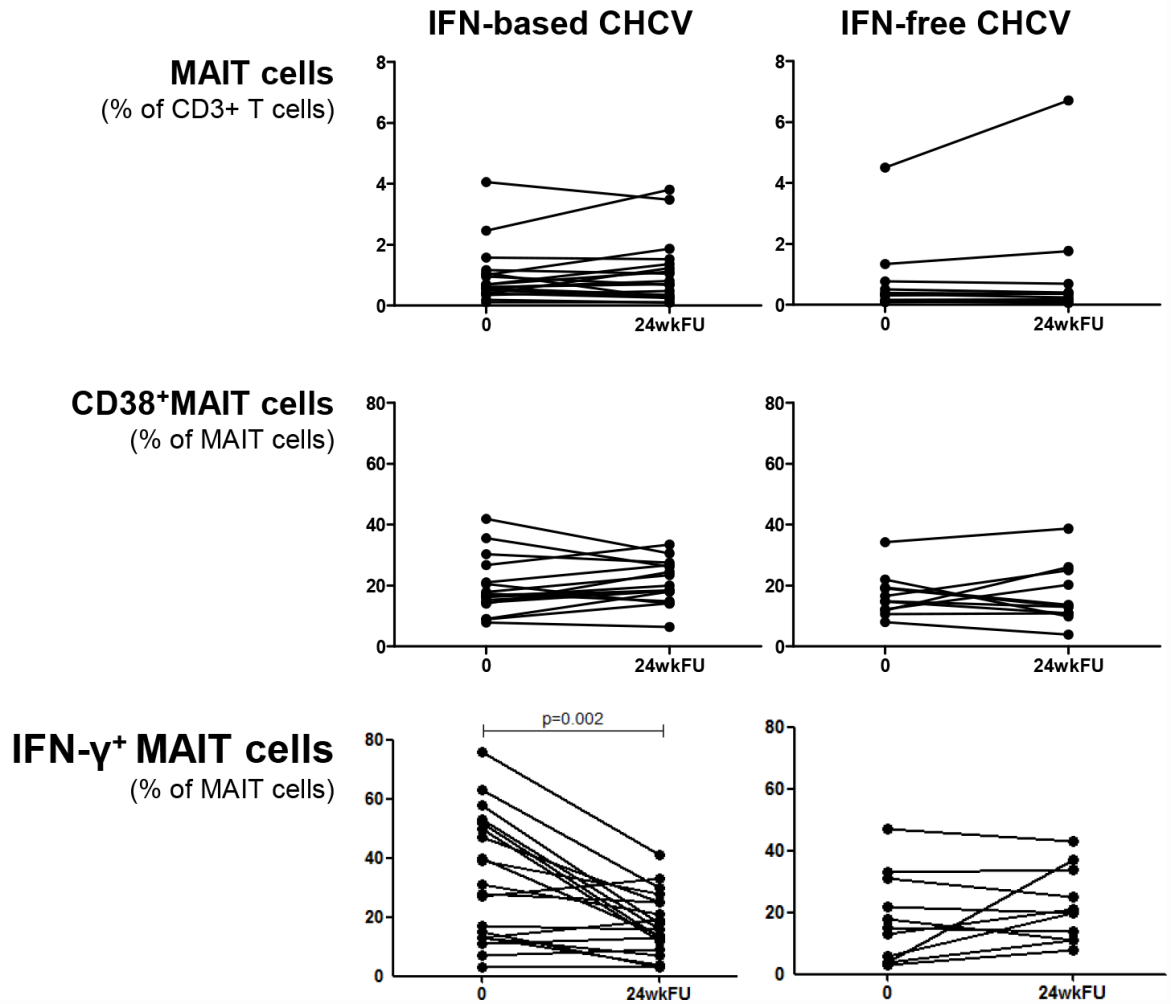
Frequency of MAIT cells in peripheral blood of chronic HCV patients does not recover after successful therapy. Frequencies of MAIT, CD38^+^MAIT and IFN-γ^+^ MAIT cells before and 24 weeks after successful IFN-based (left, n = 22) and IFN-free (right, n = 9) therapy for CHCV. Frequencies of IFN-γ^+^ MAIT cells were measured after 19 hours stimulation with IL-12/18. Statistical comparison was tested using Wilcoxon matched pairs T test.

Besides evaluation of the effect of therapy on phenotype and function of MAIT cells, it is highly relevant to determine the effect of viral clearance. Blood was collected 24 weeks after end of therapy. No samples from patients with AHCV/HIV co-infection were available after therapy. Fig 4 shows that 24 weeks after cessation of IFN-based therapy as well as IFN-free therapy, the MAIT cell frequencies were not restored in CHCV patients and remained low (Fig 4, upper panels). The increased expression of CD38 on MAIT cells during IFN-based therapy for CHCV (Fig 3A) was not maintained 24 weeks after therapy and returned to pre-treatment levels (Fig 4). Interestingly, the suppressive effect of IFN-based therapy on IFN-y producing MAIT cells as shown in Fig 3, was still observed 24 weeks after cessation of therapy upon IL-12/IL-18 stimulation (p = 0.002, Fig 4). Therefore, it appears that IFN-based therapy for CHCV affects IFN-y producing MAIT cells at least up to 24 weeks after therapy. No differences in frequencies of MAIT cells, CD38^+^ MAIT cells or IFN-γ producing MAIT cells were observed before and 24 weeks after IFN-free therapy for CHCV (Fig 4, right panels).

## Discussion

In this study we performed a detailed analysis on MAIT cells in CHCV, HIV and AHCV/HIV infections. We observed that the frequency of MAIT cells is decreased in all three groups of infected patients compared to healthy individuals, and that no normalization was observed following successful anti-HCV therapy at a follow-up period of 24 weeks. Moreover, the frequency of IFN-y producing MAIT cells upon IL-12/IL-18 stimulation was reduced in blood from HCV patients receiving IFN-α-based therapy, but not in blood from patients receiving IFN-free therapy.

Our findings on reduced MAIT cell frequencies in HIV and HCV are in line with others [15–18, 31]. We confirmed the recent study by Hengst et al. [23] by showing reduced frequencies of CD161^+^Vα7.2^+^MAIT cells in peripheral blood of CHCV patients, long after viral clearance. From our studies, we cannot conclude whether the reduced frequencies of MAIT cells are due to depletion of cells via apoptosis, migration of MAIT cells from blood to peripheral organs or skin, or due to down-regulation of characteristic markers, such as CD161. However, others have demonstrated that it is unlikely that CD161 down-regulation is responsible for the observed MAIT cell numbers in HIV infection [19]. In addition, it has been shown that MAIT cells are systemically depleted in simian immunodeficiency virus infected rhesus macaques, a model often used to investigate HIV [32]. This suggests that migration of MAIT cells to peripheral organs in HIV is less likely. More research is needed to clarify the cause of the depletion in HIV and HCV. Importantly, virus eradication by IFN-based therapy as well as by IFN-free therapy did not lead to normalization of the reduced MAIT cell frequencies at 24 weeks after cessation of therapy. These findings are similar to the observations by Hengst et al. who showed nonreversible MAIT dysfunction 48 weeks after end of treatment with sofosbuvir and ribavirin [23]. The long-term complications of low MAIT cell frequencies may be an increased susceptibility of bacterial infections after viral clearance. These observations are reminiscent of the findings reported in HIV where long-term suppression of viral replication by cART does not result in normalization of MAIT numbers in blood [15, 17, 18]. It is well-known that the immune system in cART-controlled HIV patients remains at a higher activation status as compared to control healthy individuals, but it is unknown whether the enhanced immune activation mediates MAIT cell depletion. Besides a lower frequency of MAIT cells, we also observed a lower frequency of NK cells in HIV patients compared to CHCV and healthy individuals (p = 0.02 and p = 0.02). Decreased NK cell frequencies and function in HIV patients has been described before to be associated with a more rapid progression to AIDS in untreated patients [33].

Besides their numbers, the function of MAIT cells is also an important determinant of their contribution to the overall response to HIV or HCV. As shown in this manuscript and reported by others, MAIT cells are highly responsive to IL-12/IL-18 [12, 16]. We now show that MAIT cells are also responsive to the combination of IFN-α and IL-18, leading to the production of IFN-γ. The ability of MAIT cells to respond to IFN-α is shared with NK cells.

The function of MAIT cells, as reflected by the frequency of IFN-y producing MAIT cells upon IL-12/IL-18 stimulation was similar in patients with CHCV, HIV or AHCV/HIV as compared to healthy individuals. Recently, dysfunctional MAIT cells from chronic HCV patients with respect to their ability to produce IFN-γ were observed upon MR1-dependent antigen stimulation, but -similar to our findings- not upon IL-12/IL-18 stimulation [23]. However, since MAIT cells are decreased in these patients groups, the total amount of IFN-y by these MAIT cells is likely to be decreased. Also the activation state of MAIT cells from CHCV and HIV patients were not affected. In contrast, MAIT cells from AHCV/HIV patients exhibited higher frequencies of CD38-expressing MAIT cells, which may be the result of exposure to pro-inflammatory serum cytokines that are known to be present at relatively high levels during acute HCV infection [34].

Viral load reduction by DAA-therapy in CHCV patients did not affect MAIT cell activation or IFN-γ^+^ frequencies, whereas IFN-based therapy strongly affected both parameters. We observed that IFN-based therapy enhanced the expression of the activation marker CD38 on MAIT cells, but decreased the frequencies of IFN-y producing MAIT cells. Since the activation state of MAIT cells during IFN-based therapy is augmented, it is unlikely that the effect on IFN-y production is the result of an overall inhibitory effect on the MAIT cells, but it may be the consequence of functional paralysis due to prolonged exposure to IFN-α. Although highly speculative, this may also explain the opposing effects of IFN-α on the frequencies of IFN-y producing MAIT cells upon short-term exposure *in vitro* and long-term exposure *in vivo*. The observation that IFN-based therapy, but not DAA-therapy, suppresses the functionality of MAIT cells, may indicate that a disadvantage of IFN-based-therapy over DAA therapy is that it affects the anti-microbial function of MAIT cells and that the reduced functionality of MAIT cells may render individuals more susceptible to pathogens.

In conclusion, we show that MAIT cells are decreased in patients with chronic HCV, HIV and AHCV/HIV co-infection compared to healthy controls. We show that IFN-α modulates MAIT cells *in vitro*, and *in vivo* during IFN-based therapy for HCV but importantly, a sustained viral response for HCV does not rescue MAIT cell frequency.

## Supporting Information

S1 Fig No clear differences are observed between CHCV patients treated with IFN/based therapy with or without telaprevir. Patients with CHCV were treated with peginterferon and ribavirin alone (n = 9) or in combination with telaprevir for 12 weeks (n = 11). All patients were responsive to either treatment and were HCV RNA negative at week 12 of therapy (<15 U/ml). Frequencies of MAIT, CD38+MAIT and NK cells before and 12 weeks during therapy are shown.
(TIF)
