# Supplementary figures and images for: Frequencies of Circulating MAIT Cells Are Diminished in Chronic HCV, HIV and HCV/HIV Co-Infection and Do Not Recover during Therapy
Source: PLoS One. 2016 Jul 14;11(7):e0159243. doi: 10.1371/journal.pone.0159243 (PMC4945024; doi:10.1371/journal.pone.0159243)

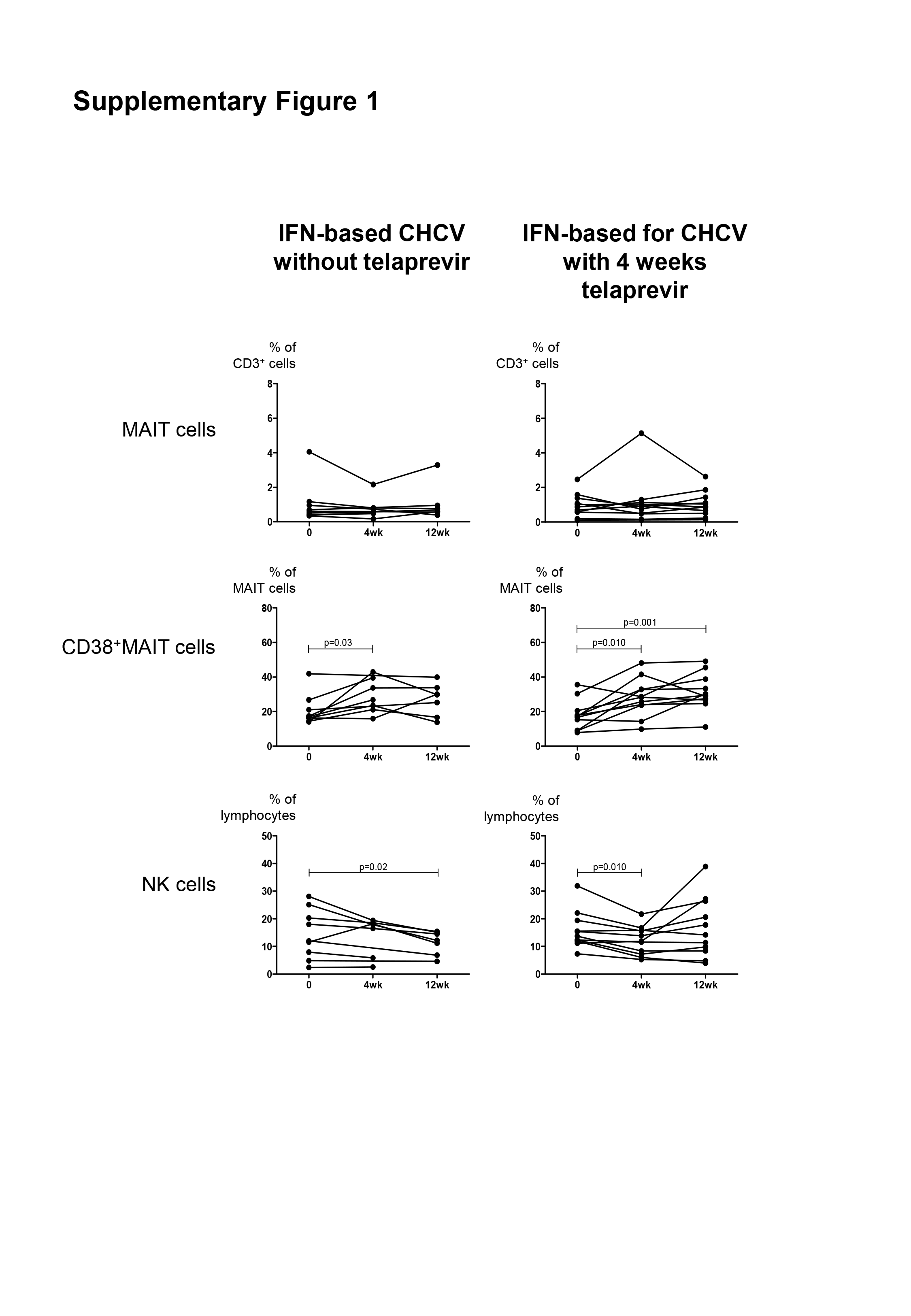

Supplement: S1 Fig — Patients with CHCV were treated with peginterferon and ribavirin alone (n = 9) or in combination with telaprevir for 12 weeks (n = 11). All patients were responsive to either treatment and were HCV RNA negative at week 12 of therapy (<15 U/ml). Frequencies of MAIT, CD38+MAIT and NK cells before and 12 weeks during therapy are shown. (TIF) [file pone.0159243.s001.tif]
